# The complete mitochondrial genome of *Sclerophytum grandilobatum* (Verseveldt, 1980) (Anthozoa: Octocorallia: Sarcophytidae) and its phylogenetic position

**DOI:** 10.1080/23802359.2026.2664910

**Published:** 2026-05-04

**Authors:** Chun-Yang Shen, Wei Xue, Qiang-Xing Shao, Hao-Yu Shi, Lu Huang, Zhao-Ying Ma, Zi-Xian Zhang, Xin-Ran Yu, Hui-Fang Guo

**Affiliations:** a Department of Biology, Chengde Medical University, Chengde, China; b Department of Chemical Engineering, Hebei Petroleum University of Technology, Chengde, China; c Institute of Sericulture, Chengde Medical University, Chengde, China

**Keywords:** Soft coral, mitogenome, *Sinularia*, *Sclerophytum grandilobatum*, phylogeny

## Abstract

Species within the genus *Sclerophytum* exhibit highly complex morphological characteristics, making morphological data alone insufficient for resolving their phylogenetic relationships; thus, molecular evolutionary data are valuable for inferring phylogenetic relationships within this genus. In the present study, the complete mitochondrial genome (mitogenome) of the soft coral *Sclerophytum grandilobatum* (Verseveldt, 1980) was sequenced and annotated. The mitogenome of *S. grandilobatum* was 18,752 bp in length and consisted of 14 protein-coding genes (PCGs), two ribosomal RNA genes (rRNAs), and one tRNA gene (*tRNA-Met*). Phylogenetic analysis confirmed the monophyly of *Sclerophytum*, with *S. grandilobatum* forming a well-supported sister lineage to all other congeners.

## 1. Introduction

A colony of *Sinularia* May, 1898 (registration no. 6500) from the Berlin Museum, collected at Puerto Galera Bay (Philippines), was initially identified as *Sinularia macrodactyla*. Subsequently, it was distinguished from *S. macrodactyla* based on differences in sclerites, and especially the clubs in the surface layer of the lobes and stalk. Based on these distinct morphological variations, Verseveldt (1980) described a new species, *Sinularia grandilobata*.

Further morphological analysis revealed that four *Sinularia* species, *S. vrijmoethi* (Verseveldt 1971), *S. grandilobata* (Verseveldt 1980), *S. loyai* (Verseveldt and Benayahu 1983), and *S. flaccida* (van Ofwegen 2008), share identical key morphological traits, including a prominent collaret and tentacle sclerites composed of points and rods. All four species have club-shaped surface sclerites with a central wart partially obscured by three lateral warts; *S. vrijmoethi*, *S. flaccida*, and *S. loyai* possess elongated club-shaped sclerites and a stalked growth form, while *S. grandilobata* has distinctly shorter sclerites and an encrusting growth form (McFadden et al. 2009).

However, morphological plasticity limits morphological identification accuracy in these species (McFadden et al. 2009, 2022). To resolve this issue, McFadden et al. (2009) reconstructed the *Sinularia* phylogeny using the mitochondrial *mutS* gene and recovered five major clades. Clade 2 forms a morphologically cohesive group with distinct collaret and tentacle sclerites, including *S. grandilobata*.

Advances in molecular phylogenetic analyses have driven major revisions in octocoral taxonomy, including the recent reclassification of the genus *Sinularia* to *Sclerophytum* (family Sarcophytidae) and *S. grandilobata* to *Sclerophytum grandilobatum* (see McFadden et al. 2022). As originally described, *S. grandilobatum* is generally characterized by its very stout lobes, which can reach up to 120 mm in length and 30 mm in width at the base (Verseveldt 1980).

Despite progress in *Sclerophytum* taxonomy and molecular research, widespread morphological plasticity and limited molecular data hinder its phylogenetic resolution. Only single-gene sequences are available for *S. grandilobatum*, whose mitogenome remains unreported; since mitogenomes yield richer data than single genes, this insufficiency constrains insights into its phylogenetic placement and the genus’ evolution.

This study entailed sequencing and annotating the mitogenome of *S. grandilobatum*, followed by phylogenetic analysis using available congeneric mitogenomes. By expanding the mitogenomic dataset of *Sclerophytum*, we aim to refine insights into the evolutionary relationships, phylogeny, and taxonomic classification of the genus, and to provide baseline data for mitogenome evolution studies of Malacalcyonacea.

## 2. Materials and methods

### 2.1. Sample collection

**Figure 1.**
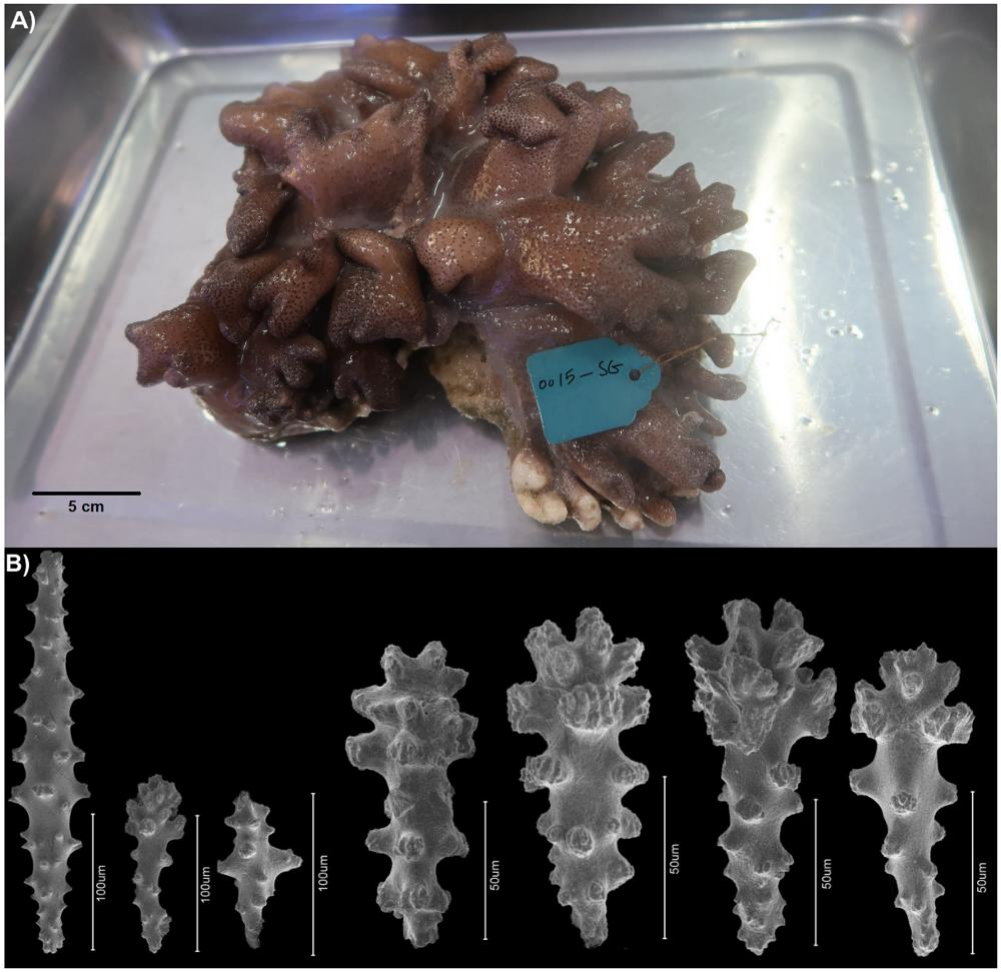
(A) Image of the live specimen of *S. grandilobatum*. (B) SEM morphology of sclerites from the colony polyps and surface of *S. grandilobatum*. Photos by Alireza Asem.

A specimen of *S. grandilobatum* was collected in June 2019 by Alireza Asem from Hainan Tropical Ocean University through scuba diving at West Island, Sanya, Hainan Province, China (18° 14′ 5.93″ N, 109° 22′ 46.46″ E). Following McFadden et al. (2009), its taxonomic status was confirmed via the morphology of sclerites. A photograph of the *S. grandilobatum* colony is shown in Figure 1, along with sclerite morphology. Both the collected colony and extracted genomic DNA have been deposited at the Hainan Tropical Ocean University Museum of Zoology (specimen voucher number: 0015-SG; DNA ID number: 0015-D; Contact: Alireza Asem, asem.alireza@gmail.com).

### 2.2. DNA extraction and sequencing

Total genomic DNA was extracted from 45 mg tissue using the Genomic Animal DNA Isolation Kit (Sangon Biotech Co., Ltd., Shanghai, China; No. B518221). A 2 × 150 bp paired-end library was constructed with the NEB Next^®^ Ultra™ DNA Library Prep Kit (New England Biolabs, Inc., Ipswich, MA; No. E7370L) and sequenced on the Illumina HiSeq X platform, yielding 12.9 Gb of raw data.

### 2.3. Genome assembly, annotation

Quality control of the raw sequencing reads was performed using FastQC (Andrews 2010), with the error rate and quality score distributions along the reads visualized in Figure S1. For *de novo* assembly, the mitogenome of *Sclerophytum ceramense* (GenBank Accession No. MK292119; see Asem et al. 2019) was used as the reference genome. *De novo* assembly was then conducted using GetOrganelle v1.7.7.0, with k-mer values set to 21, 45, 65, 85, and 105 (Jin et al. 2020).

**Figure 2.**
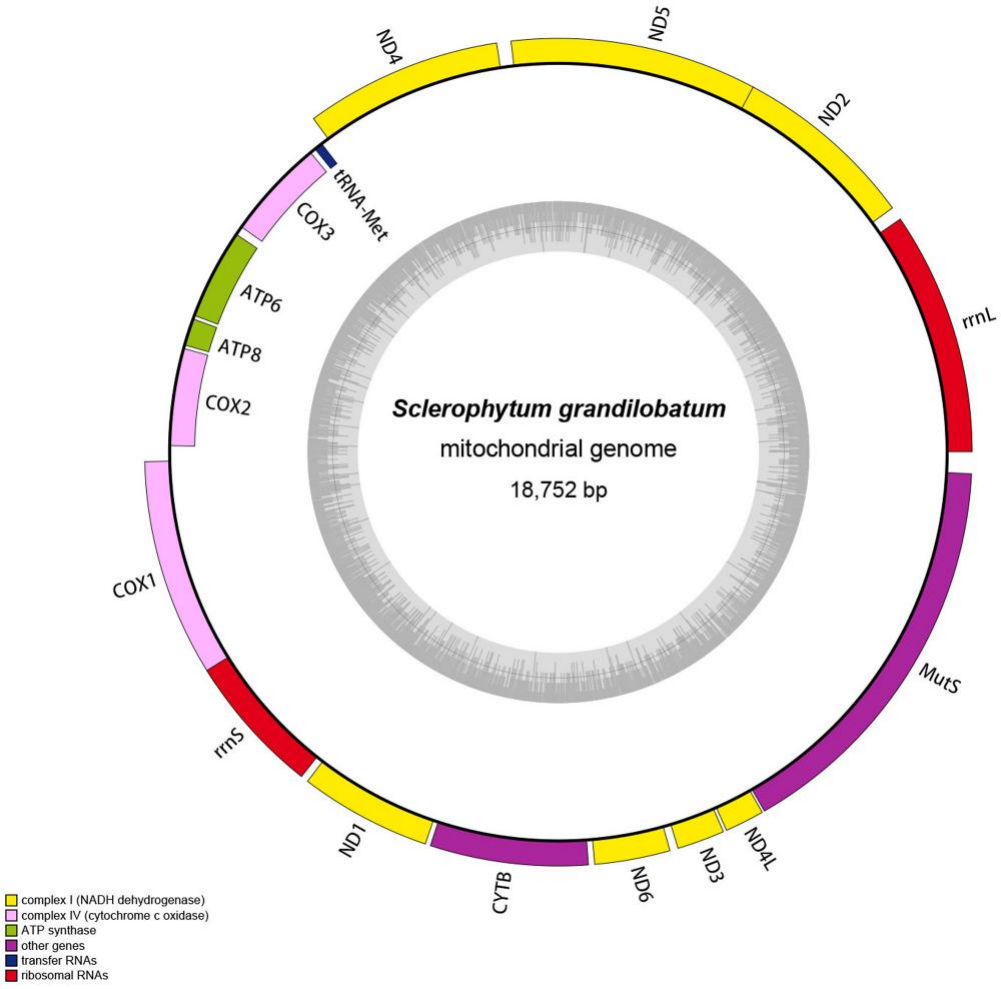
Circular map of the complete mitochondrial genome of *S. grandilobatum* (GenBank No. PX856750).

tRNA gene (tRNA) annotation was conducted using ARWEN (Laslett and Canbäck 2008). Protein-coding genes (PCGs) and ribosomal RNA genes (rRNAs) were annotated in BioEdit (Hall 1999) by alignment to the reference mitogenome. Further validation included translating each PCG into amino acid sequences (via ExPASy Translate Tool, https://web.expasy.org/translate/) to confirm start/stop codon positions and overall gene orientation. The mitogenome of *S. grandilobatum* was then mapped as a circular diagram using OGDRAW (Greiner et al. 2019) (Figure 2).

### 2.4. Phylogenetic analysis

For the phylogenetic analysis, 12 mitogenomes of the genus *Sclerophytum* were employed. Two outgroups were selected following the authoritative octocoral phylogenetic framework (McFadden et al. 2022): *Sarcophyton trocheliophorum* (Sarcophytidae, same family as *Sclerophytum*) and *Lateothela grandiflora* (Alcyoniidae, within the same major clade M3 of Malacalcyonacea). Fourteen PCGs and two rRNAs were individually aligned in MEGAX (Kumar et al. 2018), and their separate alignments were concatenated using SequenceMatrix (Vaidya et al. 2011). The best-fit Tamura–Nei + G model was selected using jModelTest (Darriba et al. 2012), and the ML tree was constructed in MEGA7 with 1000 bootstrap replicates under this model (Kumar et al. 2016).

## 3. Results

### 3.1. Mitochondrial genome characteristics

The sequencing depth results for the *S. grandilobatum* mitogenome assembly are presented in Figure S2, with a mean of 766.45× and a range from 519× to 2271×. The mitogenome of *S. grandilobatum* was 18,752 bp in length, with a base composition of 30.14% A, 16.65% C, 19.47% G, and 33.75% T.

Fourteen PCGs, two rRNAs, and one tRNA were encoded in the *S. grandilobatum* mitogenome. The *tRNA-Met* and four PCGs (*COX3*, *ATP6*, *ATP8*, *COX2*) were located on the light strand, while the remaining 10 PCGs (*COX1*, *ND1*, *CYTB*, *ND6*, *ND3*, *ND4L*, *MutS*, *ND2*, *ND5*, *ND4*) were distributed on the heavy strand (Figure 2).

All PCGs initiated translation with the canonical ATG start codon. Regarding stop codons, nine PCGs (*ND1*, *CYTB*, *ND6*, *ND3*, *ND2*, *ND5*, *COX3*, *COX1* and *COX2*) utilized TAG as their terminal codon. Five additional PCGs, including *ND4L*, *MutS*, *ND4*, *ATP6*, and *ATP8*, employed TAA as their stop codon.

### 3.2. Phylogenetic tree

**Figure 3.**
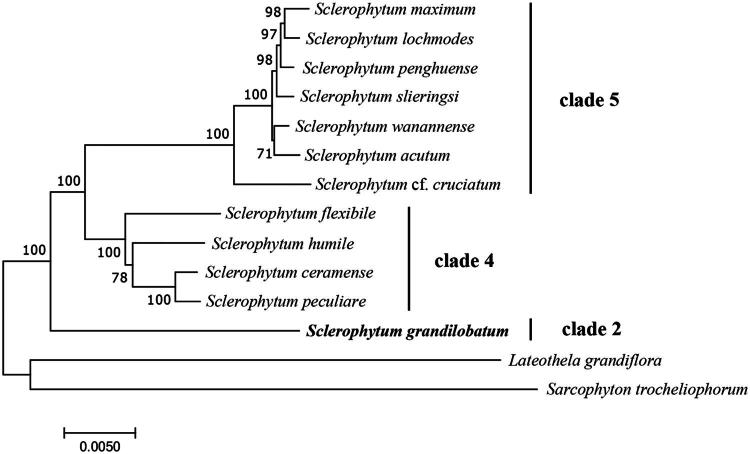
Phylogenetic tree representing the relationship of the genus *Sclerophytum* based on the concatenated nucleotides of 14 protein coding genes and two ribosomal RNA genes using maximum-likelihood (ML). The numbers of each node show the bootstrap support values. The bold indicates the species *S. grandilobatum* (PX856750; this study) sequenced in this study. The other included taxa are the following: *Sclerophytum maximum* (MN485891; Chen et al. 2019), *Sclerophytum penghuense* (MW256412; Shen et al. 2021), *Sclerophytum acutum* (MW987591; Yang et al. 2023), *Sclerophytum* cf. *cruciatum* (KY462727; Shimpi et al. 2017), *Sclerophytum humile* (OK641586; Yang et al. 2022), *S. ceramense* (MK292119; Asem et al. 2019), *Sclerophytum peculiare* (JX023274; Kayal et al. 2015), *Sclerophytum flexibile* (OL616272; Muthye et al. 2022), *Sclerophytum lochmodes* (OL616273; Muthye et al. 2022), *Sclerophytum slieringsi* (OL616276; Muthye et al. 2022), *Sclerophytum wanannense* (OL616277; Muthye et al. 2022), *S. trocheliophorum* (MK994517; Shen et al. 2019), and *L. grandiflora* (PX070533; Quattrini et al. 2024), with the latter two serving as outgroups.

The phylogenetic tree (Figure 3), constructed via ML, showed strong nodal support across the topology. The phylogenetic tree placed *S. grandilobatum* as the sister lineage to other *Sclerophytum*, and the genus formed a strongly supported monophyletic clade.

## 4. Discussion and conclusions

Comparison of gene order between *S. grandilobatum* and all available *Sclerophytum* mitogenomes (see Kayal et al. 2015; Shimpi et al. 2017; Asem et al. 2019; Chen et al. 2019; Shen et al. 2021; Muthye et al. 2022; Yang et al. 2022, 2023) revealed identical arrangement patterns. This highly conserved architecture conforms to the ancestral pattern A of Malacalcyonacea defined by Yoshioka et al. (2025), the dominant gene order within Sarcophytidae.

The phylogenetic tree confirmed the monophyly of *Sclerophytum*, resolving into three major clades. Species composition within each clade matched that of McFadden et al. (2009), except for taxa absent from their sampling. Following their topology, the three recovered lineages were designated clades 2, 4, and 5. *S. grandilobatum* was recovered as the basal sister lineage to all congeners. Additional mitochondrial genomes from *Sclerophytum*, especially lineages outside these three clades, are required to clarify their maternal phylogenetic relationships.

This study first reports the mitogenome of *S. grandilobatum*. We characterized its genomic features and phylogenetic relationships, providing molecular support for future phylogenetic, population genetic, and evolutionary studies of *Sclerophytum*. Further mitogenome sequencing of *S. macrodactyla—*morphologically similar to *S. grandilobatum—*will help resolve morphological and molecular congruence within the genus.

## Supplementary Material

Supplemental Material

Supplemental Material

## Data Availability

The mitogenome sequence data that support the findings of this study are available in GenBank of NCBI at https://www.ncbi.nlm.nih.gov/ under the accession no. PX856750 (https://www.ncbi.nlm.nih.gov/nuccore/PX856750). The associated BioSample, BioProject, and SRA numbers are SAMN49820411 (https://www.ncbi.nlm.nih.gov/biosample/SAMN49820411), PRJNA1287474 (https://www.ncbi.nlm.nih.gov/bioproject/PRJNA1287474), and SRR34411016 (https://www.ncbi.nlm.nih.gov/sra/SRR34411016), respectively.
