# Supplementary figures and images for: The complete mitochondrial genome of Sclerophytum grandilobatum (Verseveldt, 1980) (Anthozoa: Octocorallia: Sarcophytidae) and its phylogenetic position
Source: Mitochondrial DNA B Resour. 2026 May 4;11(6):708–11. doi: 10.1080/23802359.2026.2664910 (PMC13142183; doi:10.1080/23802359.2026.2664910)

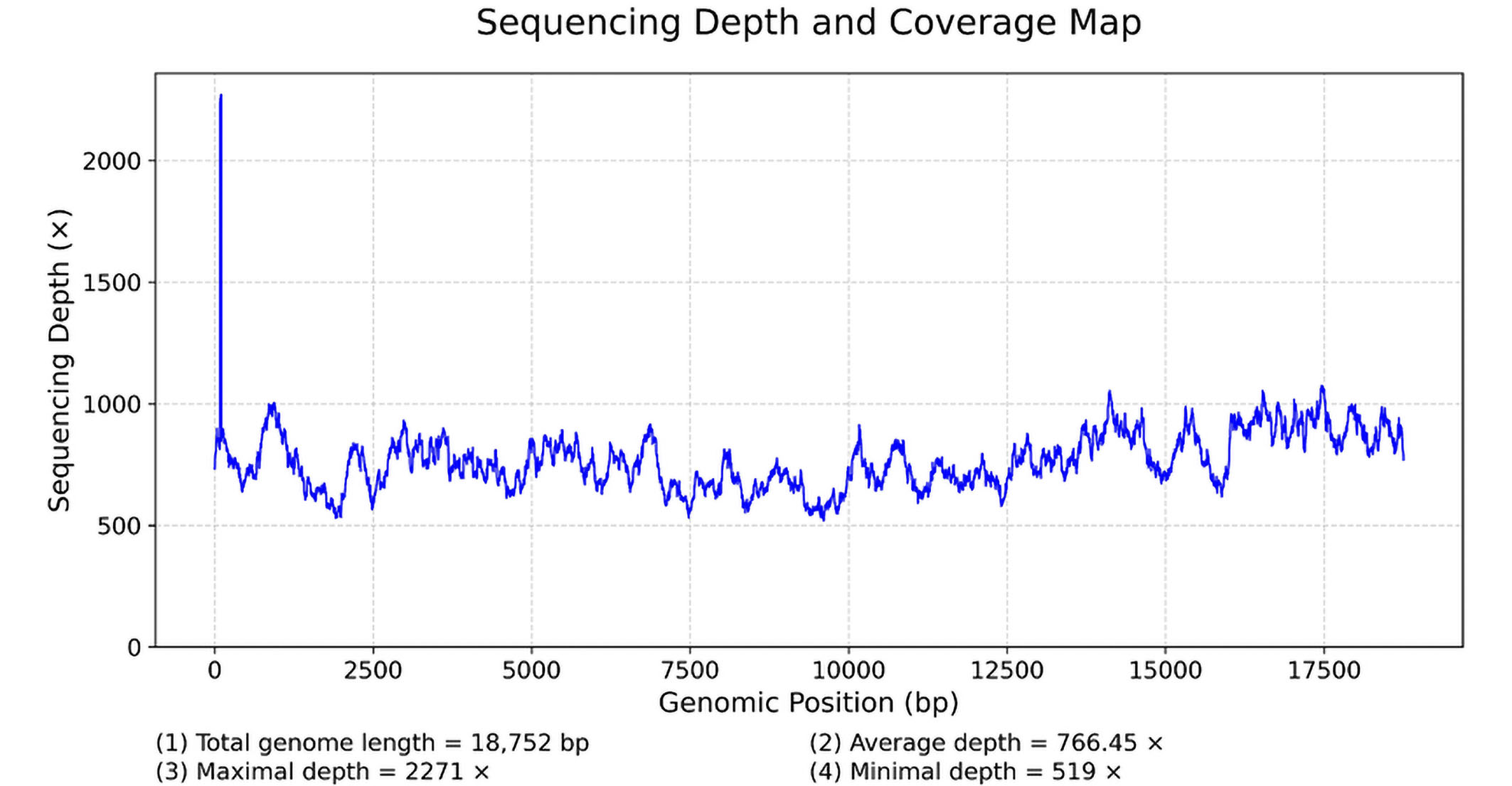

Supplement: Supplemental Material [file TMDN_A_2664910_SM6657.jpg]

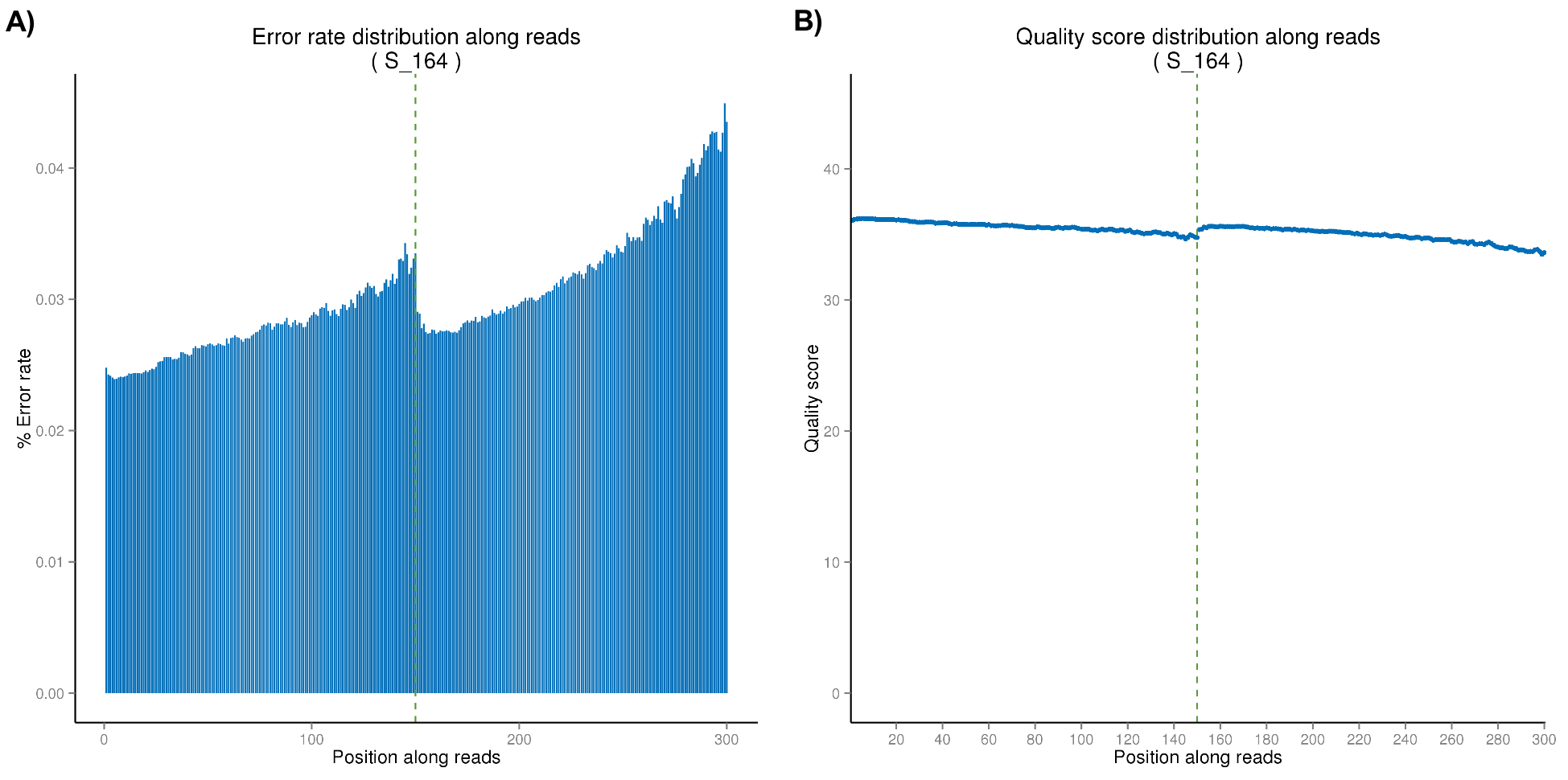

Supplement: Supplemental Material [file TMDN_A_2664910_SM6654.jpg]
